# Combining biological motion perception with optic flow analysis for self-motion in crowds

**DOI:** 10.1167/jov.20.9.7

**Published:** 2020-09-09

**Authors:** Anna-Gesina Hülemeier, Markus Lappe

**Affiliations:** Department of Psychology, University of Münster, Münster, Germany

**Keywords:** biological motion, optic flow, heading

## Abstract

Heading estimation from optic flow relies on the assumption that the visual world is rigid. This assumption is violated when one moves through a crowd of people, a common and socially important situation. The motion of people in the crowd contains cues to their translation in the form of the articulation of their limbs, known as biological motion. We investigated how translation and articulation of biological motion influence heading estimation from optic flow for self-motion in a crowd. Participants had to estimate their heading during simulated self-motion toward a group of walkers who collectively walked in a single direction. We found that the natural combination of translation and articulation produces surprisingly small heading errors. In contrast, experimental conditions that either present only translation or only articulation produced strong idiosyncratic biases. The individual biases explained well the variance in the natural combination. A second experiment showed that the benefit of articulation and the bias produced by articulation were specific to biological motion. An analysis of the differences in biases between conditions and participants showed that different perceptual mechanisms contribute to heading perception in crowds. We suggest that coherent group motion affects the reference frame of heading perception from optic flow.

## Introduction

Locomotion through the environment generates a pattern of visual motion on the retina called optic flow (Gibson, 1950). The optic flow is a source of information for the perception of the direction in which one is heading (Bruss & Horn, 1983; Longuet-Higgins & Prazdny, 1980). The accuracy of heading perception from optic flow ranges within 1° to 2° of visual angle (Royden, Banks, & Crowell, 1992; Warren, Morris, & Kalish, 1988), sufficient for safe navigation (Cutting, 1986; Cutting, Springer, Braren, & Johnson, 1992). The visual system maintains heading accuracy even when eye movements add rotational components to the optic flow field on the retina (Li & Warren, 2000; Royden, Banks, & Crowell, 1992; van den Berg, 1993; Warren & Hannon, 1990). To perceive heading accurately and robustly, studies suggest that spatial pooling over a large part of the visual field is important (Andersen & Saidpour, 2002; Koenderink & van Doorn, 1987; Lappe & Rauschecker, 1993). Computational models propose that the visual system extracts relevant information for heading from the global structure of the optic flow field, and accounts for translational and rotational components of self-motion in a static environment (Beintema & van den Berg, 1998; Lappe & Rauschecker, 1993; Perrone & Stone, 1994).

The assumption of a static environment is central for the computational analysis of optic flow. When this assumption is violated, for example, when objects move independently in the world, heading perception becomes biased (Layton & Fajen, 2016a; Li, Ni, Lappe, Niehorster, & Sun, 2018; Royden & Hildreth, 1996; Warren & Saunders, 1995). The bias is in accordance with the most likely heading computed from the global flow field under the assumption of observer translation and rotation in a static environment (Li, Ni, Lappe, Niehorster, & Sun, 2018).

The most extreme violation of the assumption of a static environment occurs when all visible objects move independently. Strikingly, this is a common situation when one moves through a crowd of people, like in a busy train station, for example. Riddell and Lappe (2018) recently conducted a series of experiments to investigate the ability to estimate the heading of self-motion through a crowd of walkers. They found, as expected, elevated levels of heading error compared to a static world, but also that the motion of the walkers in the crowd, that is, biological motion (Johansson, 1973), contains cues useful for heading estimation.

Biological motion refers to the movements of the limbs during walking. It is characterized by an articulation and a translation component. Articulation refers to the relative change of the joint positions to each other (Blake & Shiffrar, 2007; Johansson, 1973; Masselink & Lappe, 2015). Translation refers to the linear progressive motion of the body through space (Blake & Shiffrar, 2007; Masselink & Lappe, 2015; Riddell & Lappe, 2018). In natural locomotion, articulation and translation are linked such that the articulation delivers cues about the speed and direction of the walker (Giese & Lappe, 2002; Masselink & Lappe, 2015; Thurman & Lu, 2016).

Riddell and Lappe (2018) aimed to determine the influence of articulation and translation on heading perception in a crowd. They used stimuli consisting of eight point-light walkers, which walked in random, but overall balanced, directions. Further, they used three different walker types (normal walkers, inverted walkers, and spatially scrambled nonbiological stimuli) to test for the impact of biological motion and the human figure. Last, they used four different combinations of articulation and translation. In one condition, walkers articulated and translated normally through the world. In another condition, walkers only articulated but did not translate, like walking on a treadmill. In a third condition, walkers only translated but did not articulate, like a figure skater. In a fourth condition, walkers neither articulated nor translated but simply remained standing in a particular posture in a particular place. This last condition forms a static environment with no independent motion and provided a baseline condition for undisturbed optic flow analysis. Heading errors were consequently the smallest in this condition. The other conditions, which contained independent motion in the form of articulation, translation, or both, produced larger heading errors. However, the natural articulation-plus-translation condition produced smaller heading errors than the only-translation condition, showing that some aspect of the articulation helped to decrease the error induced by the translation. Surprisingly, further experiments showed that this was not due to the human figure or biological motion perception per se, because similar decreases in heading errors were seen also in the inverted or nonbiological scrambled figures. Instead, the essential cue was provided by brief phases in the joint motion during which a single joint would be transiently static in the environment, such as, for example, the foot when it touches the ground. For optic flow analyses, these stable phases during normal gait provide brief windows into static aspects of the environment that are not present in the only-translation condition or the articulation in place condition. Thus, Riddell and Lappe (2018) concluded that biological motion contains an invariant cue to self-motion perception that is used in optic flow analysis.

In the crowd stimuli of Riddell and Lappe (2018), the translation directions of the walkers were balanced such that no overall left or right translation of the crowd would bias heading. However, in an experiment with only a single walker, heading perception was biased by the walker's translation (Riddell & Lappe, 2017). In this experiment, participants had to estimate the direction of heading toward a single point-light walker, which itself walked along an angled path toward the observer. The perceived heading was consistent with the vector average of the walker's translation and the observer's true self-motion. This finding would be expected if the optic flow system, like in the case of independently moving objects (Layton & Fajen, 2016a; Li, Ni, Lappe, Niehorster, & Sun, 2018; Royden & Hildreth, 1996; Warren & Saunders, 1995), did not take biological motion into account, but rather treated the entire scene as if it would arise from a static world. Recent experiments using a walker embedded in an optic flow field, similar to the typical paradigm for independent object motion, reinforce this view (Riddell, Li, & Lappe, 2019).

In the present study, we examined whether biases of heading perception occur when moving through a crowd of point-light walkers that all walk in the same direction, leading to an overall translation of the crowd relative to the observer. We combined articulation and translation in different ways to investigate how the direction of heading error is influenced by the direction of translation and articulation of the crowd.

## Experiment 1

### Methods

#### Sample

Twenty-one participants (eight males, 13 females) from the University of Münster took part in the study. Age ranged from 19 to 28 years (*M* = 22.33, *SD* = 2.29). All participants were naïve regarding the aim of the experiment. They all had normal or corrected-to-normal visual acuity. All participants gave written informed consent. Ethical approval was obtained from the ethics board of the Department of Psychology and Sport Science at the University of Münster. Participation was voluntary, anonymous, and compensated by course credits.

#### Setup

Experimental testing took place in a quiet, darkened room. Stimuli were generated using MATLAB (version R2014b, The MathWorks, Natick, MA) with the Psychophysics Toolbox V3 (Kleiner, Brainard, & Pelli, 2007) and the OpenGL libraries (version 2.1) add-ons. Stimuli were projected onto a 250 cm × 200 cm backlit screen by a VDC Display Systems Marquee 8500 projector connected to an Apple MacBook Pro or an HP Laptop Model Spectre (both equipped with a 512MB Intel HD graphic card). Screen resolution was 800 × 600 pixels with a frame rate of 120 Hz. Participants sat 100 cm away from the screen on a chair, resulting in a visual field of 102° × 90°. They registered their responses by moving a cursor and pressing the left button of a computer mouse. The resolution limited the response accuracy to about 0.1° of visual angle.

#### Scene

Most experimental details followed Riddell and Lappe (2018). The scene comprised eight life-sized (182 cm in height) point-light walkers. The point-light walkers were derived from the motion tracking data of a single walking human (de Lussanet, Fadiga, Michels, Seitz, Kleiser, & Lappe, 2008). Each point-light walker consisted of 12 white points corresponding with the left and right ankle, knee, hip, hands, elbow, and shoulder joints. They were located on an invisible ground plane that constituted a world coordinate system with a depth of 20 m. The ground plane was 140 cm below the eye height of the seated observer to match the ground in the experimental room. The translation speed of a walker was 0.6 m/s. The walkers started from different phases in the gait cycle.

**Figure 1.**
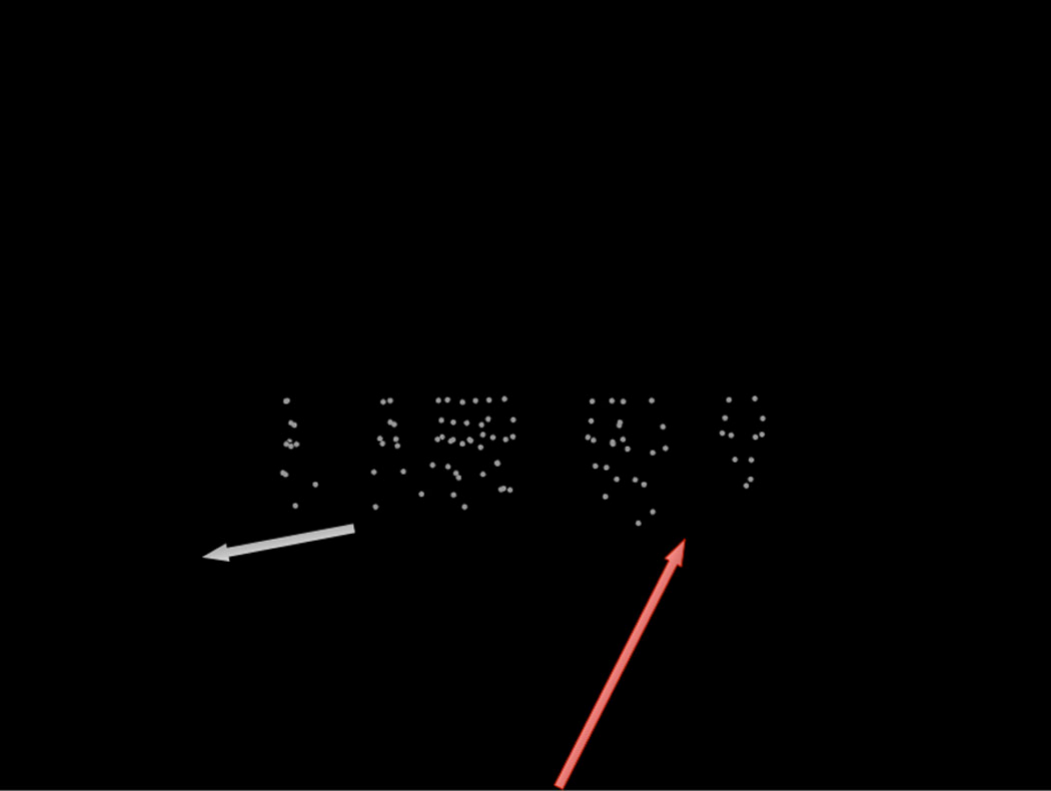
Single frame of the stimulus with a crowd of forwards moving point-light walkers. The stimulus consisted of a group of point-light walkers walking coherently into a common direction simultaneously with simulated forward movement of the observer. The *white arrow* (*left*) indicates the direction of movement of the point-light walkers in this example. The *red arrow* (*right*) indicates the simulated self-motion of the observer.

The point-light walkers were scaled with depth to appear at an appropriate retinal size for the observer. The stimulus simulated self-motion through the world at a speed of 1.1 m/s in a randomized heading direction between –12° and 12° of visual angle from the screen center. At the beginning of each trial, the walkers were placed between 27.3 m and 29.0 m in depth from the observer and between 0.55 m and 2.60 m left or right from the straight-ahead direction. This factor ensured that the full crowd would be visible on the screen throughout the trial. The points were neither transparent nor did they disappear when a walker stood behind another walker. It happened that the walkers partly overlapped (see Figure 1).

All walkers in the crowd faced in the same direction. Facing 0° was straight toward the observer and 180° corresponded with facing away from the observer. Positive facing angles marked facings to the right, and negative ones facing to the left. We presented a total of different 24 facing directions at 15° intervals.

#### Conditions

We measured perceived heading error as a function of facing direction of the group of walkers in four conditions. In the first condition, the walkers stood in place in a fixed posture in the world coordinate system. We refer to this stimulus as the static condition. In the second condition, the walkers walked across the invisible ground plane (the world coordinate system) in the direction they were facing. We refer to this as the natural articulation-plus-translation condition. In the third condition, the walkers translated across the invisible ground plane (the world coordinate system) but did not articulate their limbs, that is, they maintained a single posture. We refer to this condition as the only-translation condition. In effect, the display in this condition was equivalent to the static condition if the simulated heading was the vector sum of the observer translation and the inverse of the crowd translation. In the fourth condition, the walkers articulated their limbs but did not translate through the world, but kept a constant position in the world coordinate system, as if walking on a treadmill. We refer to this condition as the only-articulation condition. All facing directions were combined with all four conditions, resulting in a total of 96 trials within one block. The whole experiment comprised 10 blocks. The presentation of each stimulus combination was randomized within each block.

Figure 1 depicts a single frame of the stimulus with a crowd of forwards moving point-light walkers facing to the left. The translation direction of the walkers is indicated by the white arrow. The observer's simulated heading direction is marked in by the red arrow.

#### Procedure

The task of the participants was to report the perceived direction of heading. They were informed in writing and orally about the stimuli and task. We described the stimulus as a crowd of light-point walkers, who faced in different directions, for example, with their body directly toward the participant, or turned more to the left or right, or even away from the participant. In some trials, the walkers would move their limbs, in other trials they would not. This condition meant that the walkers would look different in each trial. Sometimes the walkers would move forward in the direction they are looking, and sometimes they stand or walk on the spot, like on a treadmill. We then explained to the participant that, in addition to the movement of the walkers the display also included a simulated forward motion of her- or himself that could be slightly to the left or right. We used Figure 1 to illustrate the difference between heading and the motion of the crowd.

We explained to the participant that the task was to determine the direction of this self-motion (heading) and to indicate with a mouse cursor the point on the screen in which they felt they were moving. This point could be exactly straight ahead or slightly to the left or right. The mouse cursor appeared after the end of the motion display. After the instruction, participants completed one practice block without data collection and performance feedback. The practice block contained all stimulus characteristics and combinations like an experimental block.

Afterward, the experiment started. In each trial, the scene appeared and immediately began to move. This motion lasted 2500 ms, after which the last frame remained static and a red vertical probe line appeared on the horizontal midline of the display. Participants moved the probe to their perceived heading direction and registered their response by clicking the left mouse button. Response time was not limited. The subsequent trial started directly after the response. The entire experiment took about 1.5 hours, including a short break in the middle.

Participants completed 10 blocks of data collection, each of which contained all combinations of stimulus characteristics in randomized order. Owing to technical issues during data collection, one block was lost for two participants. For these two participants, data from the remaining nine blocks were used.

### Results and discussion

#### Baseline heading performance in the static condition

First, we analyzed the heading error in the static condition in which the stimuli contained neither articulation nor translation. This condition provides a measure of baseline performance in an environment that does not contain any independent object motion. Because there was neither translation nor articulation, data were collapsed across all facing directions. One participant showed an absolute (unsigned) error of more than 3 standard deviations away from the mean, and hence was considered an outlier and removed from further data analysis. For the remaining sample of 20 participants (seven males, 13 females), the mean unsigned heading error was 2.24°, showing a good overall performance. Because our main interest in this article is any bias produced by the collective facing of the walkers in the crowd, we next analyzed the signed error. The mean signed heading error was –0.49°, showing no overall bias in the static condition.

#### Absolute heading errors for combinations of translation and articulation

In the next step, we checked whether the four conditions of translation and articulation produced results consistent with those of Riddell and Lappe (2018). They previously found that the absolute (unsigned) heading error in the articulation-plus-translation condition was smaller than in the only-translation condition.

**Figure 2.**
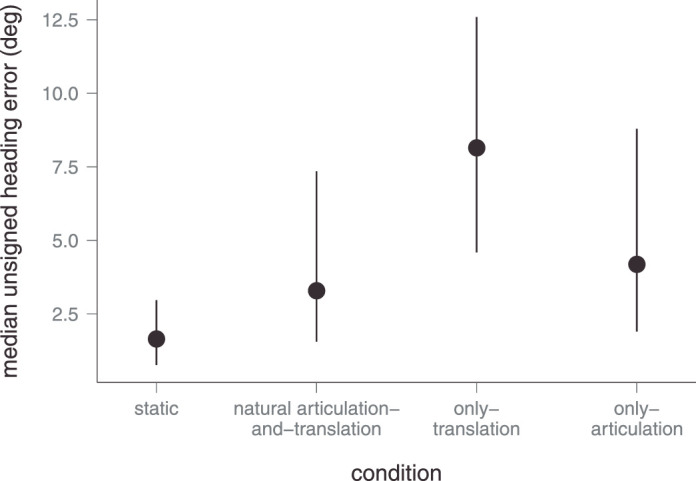
Median unsigned heading errors across all subjects and facing directions for each experimental condition. Error bars give the upper and lower interquartile ranges.

Figure 2 shows the median unsigned heading errors for each condition. Anderson-Darling Tests indicated that the data were not normally distributed, neither across conditions, *A* = 118, *p* < 0.001, nor in each condition separately, *A* ≤ 6.68, *p* < 0.001. Levene's test for homogeneity of variances showed that variances between different conditions were distributed unequally, *F*(3, 1916) = 51.02, *p* < 0.001. Thus, we conducted a Friedman rank-sum test to test differences in heading errors between conditions. Results revealed statistically significant differences between the median unsigned heading errors among conditions, χ^2^(3, 20) = 452, *p* = 0.001. Pairwise Nemenyi post hoc test for multiple comparisons showed no difference between the natural articulation-plus-translation condition and the only-articulation condition, *p* = 0.840, but highly significant differences between all other conditions, *p* < 0.001. These results replicate the findings of Riddell and Lappe (2018), providing evidence that the four conditions produce different unsigned heading errors and, in particular, that the heading error was significantly lower in the natural articulation-plus-translation condition, compared with the only-translation condition, *p* < 0.001.

#### Heading bias in the natural articulation-plus-translation condition

We next asked whether the natural articulation-plus-translation condition produced a bias similar to that observed for a single walker in the study of Riddell and Lappe (2017). Their results showed that the facing direction of a single walker significantly biased heading estimation. To investigate whether or not the articulation-plus-translation condition produced a heading bias similar to Riddell and Lappe (2017), we compared the medians of the signed heading error for the walker facings of –15°, 0°, and 15°, that is, the ones closest to the experimental setting of Riddell and Lappe (2017). In their experiment, the walker either directly approached the observer or faced 5° rightwards or leftwards, producing a bias opposite to the facing direction.

**Figure 3.**
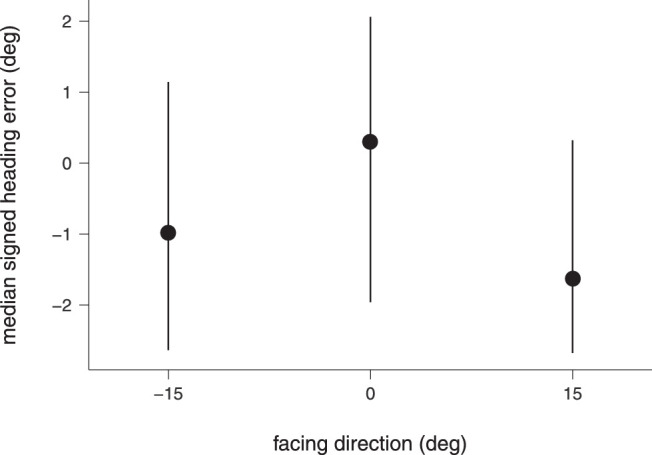
Median signed heading errors in the natural articulation-plus-translation condition for facing directions near the straight ahead (0°). Positive values indicate a heading bias to the right and negative values indicate a heading bias to the left. Error bars give the upper and lower interquartile ranges.

Figure 3 shows medians and interquartile ranges of the signed heading errors for the –15°, 0°, and 15° facings of the present data set. Results of the Anderson-Darling test revealed that data were not normally distributed, *A* = 1.439, *p* < 0.001. The Friedman rank-sum test reported no statistically significant differences between the facings, χ^2^(2, 20) = 3.10, *p* = 0.212. This finding is different from the findings of Riddell and Lappe (2017) for a single walker and provides evidence for the notion that an increased number of walkers in a crowd reduces heading errors (Riddell & Lappe, 2018).

**Figure 4.**
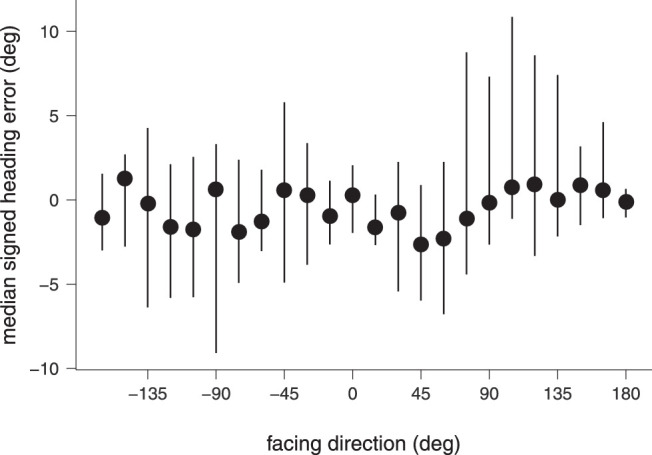
Median signed heading errors in the natural articulation-plus-translation condition for all facing directions. Error bars give the upper and lower interquartile ranges.

We then looked at this question in the broader context of all 360° facing directions in the natural articulation-plus-translation condition and asked whether there is a dependency of heading error on the direction of the crowd motion for larger discrepancies between crowd facing and observer translation. Figure 4 shows that the median signed heading error was similar across all facing directions. The data did not fulfill the requirements of a one-way analysis of variance (Anderson-Darling test), *A* = 43.73, *p* < 0.001. The Friedman rank-sum test showed no significant dependence on facing direction, χ^2^(23, 20) = 32.106, *p* = 0.098.

**Figure 5.**
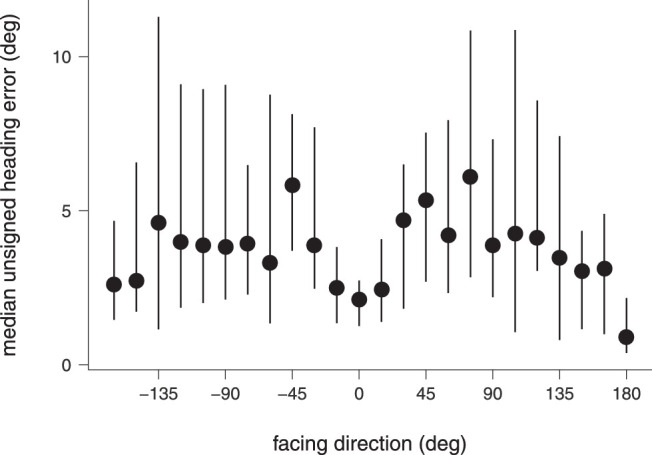
Median unsigned heading errors in the natural articulation-plus-translation condition for all facing directions. Error bars give the upper and lower interquartile ranges.

These results show that heading perception in the natural condition is remarkably robust. Yet, the error bars in Figure 4 suggest that heading estimates were more variable near the 90° facing directions when the crowd moved orthogonally to the observer. This result would predict a larger absolute heading error in these conditions. Indeed, the Friedman rank-sum test showed a significant dependence of unsigned heading error on facing, χ^2^(23, 20) = 76.03, *p* ≤ .001 (Figure 5).

**Figure 6.**
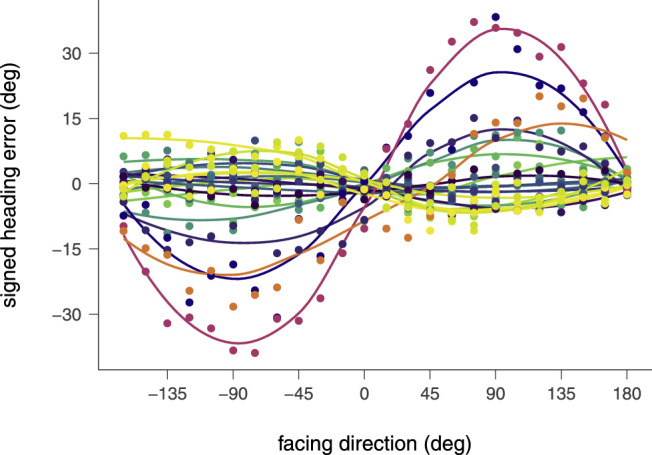
Signed heading errors of all individual participants in the natural articulation-plus-translation condition. Points represent individual means over all trials for a particular facing. Curves present local regressions through individual the data. Participants are color coded.

Individual data of the signed error is shown in Figure 6. Participants are color coded. Dots show average errors for each participant and facing. Lines depict nonparametric approximations by local regression for each participant. Figure 6 shows that most participants exhibit only small variations of their signed errors with facing direction, consistent with the small median error overall. A small number of participants, however, showed strong and systematic errors that reached up to 30° and were directed in the facing direction of the group.

#### Relation of bias in the natural articulation-plus-translation condition to biases in the only-translation and the only-articulation conditions

The articulation-plus-translation condition contained both aspects of biological motion. We wondered how these two components combine to produce the overall correct heading estimates in the combined conditions, and whether the strong errors shown by some individuals may be related to their perception of one or both of those aspects. Therefore, we next analyzed the data from the only-translation and the only-articulation conditions.

We first undertook an inferential analysis to examine how translation and articulation contribute to the variance in the articulation-plus-translation condition using a regression model in which the signed heading error in each condition is predicted by the lateral (sideways) and the longitudinal (toward/away from observer) components of the facing direction. The lateral component was calculated by the sine of the facing angle, and the longitudinal direction was calculated by the cosine. The beta weights of the sine component turned out to be a statistically significant predictor across conditions (*p* < 0.005). The cosine component had no statistically significant impact on the model in any experimental condition (*p* > 0.06) and was not considered further for the regression. Hence, the regression model delivered the intercept with the beta weight of the sine component of each subject and each condition. To examine the performance in the natural articulation-plus-translation condition in relation to its translation and articulation components, we predicted the sine component of the natural facing plus articulation condition by the sine components of the only-articulation and the only-translation condition of each participant. This process analysis gave the beta weights as standardized regression equation, *F*(2, 17) = 153.20, *p* < 0.001, with an *R^2^* of 94.74%. It confirmed that the sine components of the signed heading errors in the only-articulation condition, β = 0.66, *p* < 0.001, significantly predicted the ones in the natural facing plus articulation condition, as did the sine components in the only-translation condition, β = 0.42, *p* < 0.001.

To exclude an influence of the stimulus characteristics in the static condition on the heading errors, we repeated the above analysis with the sine components of the static condition added to the model. Results showed no significant impact of the static condition, β = –0.04, *p* = 0.745. Integrating this additional factor did not achieve a statistically significant improvement as confirmed by an analysis of variance comparing the fits of both models, *F*(1, 1) = 0.155, *p* = 0.699. Thus, the more complex model was not significantly better at capturing the data than the simpler one. Accordingly, we concluded that the simpler model with only two independent variables was preferable. Thus, our inferential analysis lent support to view that the results in the natural articulation-plus-translation condition are well-explained by the combination of translation and articulation.

#### Idiosyncratic biases in the only-translation condition

To better understand the respective contributions of the two components, we next analyzed the data of each condition separately in detail. We begin by describing the only-translation condition. In the only-translation condition, the walkers of the crowd each maintained a single static posture as they all moved in the same direction in the world coordinate system. Because they did not articulate there was no biological motion information about their translational movement. Therefore, the stimulus contained no information to separate the motion of the crowd from the self-motion of the observer. We thus expect that, because participants estimate heading from this stimulus as if it were resulting from pure self-motion, the reported heading would present a strong bias.

Indeed, a Friedman rank-sum test confirmed that the heading error depended significantly on the facing direction, *A* = 1.02, *p* < 0.01; χ^2^(23, 20) = 193, *p* < 0.001. To better understand the source of the heading bias we compared it with a simple prediction, namely that the visual system treats all image motion as the result of pure linear self-motion and computes heading perfectly under this assumption (Riddell & Lappe, 2017). Under this prediction, the perceived heading is the vector sum of the self-motion and the inverse of the translation of the crowd.

**Figure 7.**
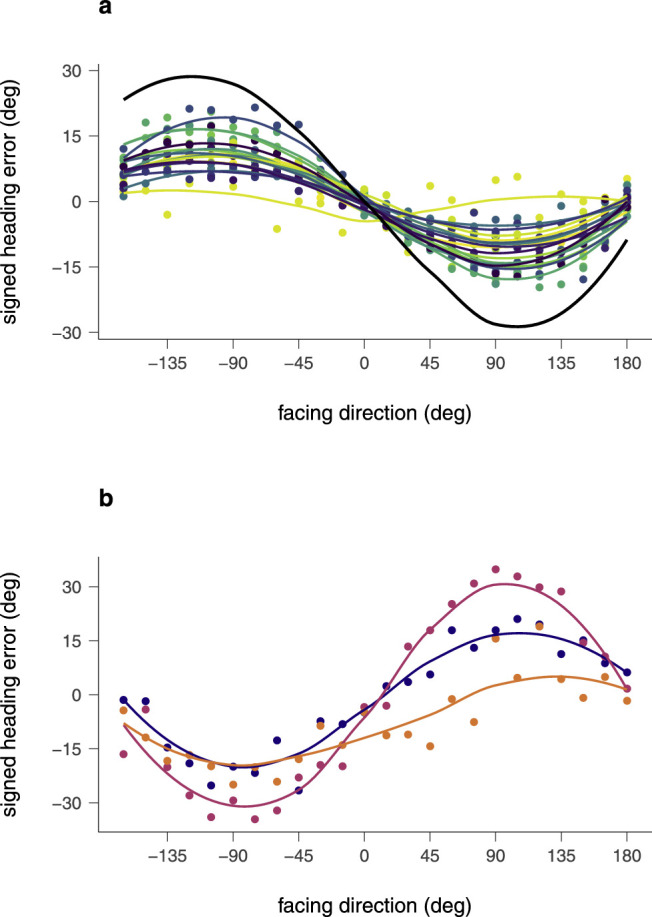
Signed heading errors of individual participants in the only-translation condition. According to the direction of bias in this condition participants were separated into two groups. (a) Participants (*n* = 17) of group A show a heading bias against the direction of group motion, that is, positive and to the right for leftward (negative) facing directions and negative and to the left for rightward (positive) facing directions. The *thick black line* shows a prediction if participants simply responded as if all motion was due to self-motion, and hence, reported the vector average of the true heading and the inverse of the translation of the group. (b) Participants (*n* = three) of group B instead displayed an opposite behavior, that is, a bias in the direction of crowd motion.

For the majority of the participants, *n* = 17, the direction of their heading bias followed this prediction (Figure 7a). The figure shows large heading errors to the right for facings to the left and the left for facings to the right. The Friedman rank-sum test confirmed a relationship between the size of the heading error and the facing direction, *A* = 1.34, *p* < 0.002; χ^2^ (23, 17) = 192, *p* < 0.001. The figure also shows that the magnitude of the heading bias is different between participants and smaller than the prediction (thick black line in Figure 7a). This smaller magnitude may be explained by a center bias that is commonly found in heading estimation from optic flow (Royden, Banks, & Crowell, 1992; van den Berg, 1993; Warren & Hannon, 1988) and in other scene-based experiments (Buswell, 1935; Itti, 2004; Foulsham, Walker, & Kingstone, 2011; Parkhurst, Law, & Niebur, 2002).

Three participants showed a striking pattern of heading errors opposite to that of the main group of participants (Figure 7b). In their case, the data showed a strong and highly significant bias to the left for facings to the left and the right for facings to the right, that is, a bias in the direction of the crowd motion, *A* = 0.55, *p* = 0.144; *F*(1, 70) = 71.55, *p* < 0.001.

Previous experiments on heading perception in the presence of independent motion have also found biases in (Li, Ni, Lappe, Niehorster, & Sun, 2018; Riddell & Lappe, 2018; Royden & Hildreth, 1996) as well as against (Layton & Fajen, 2016a; Li, Ni, Lappe, Niehorster, & Sun, 2018; Warren & Saunders, 1995) the direction of independent motion, depending on features of the stimulus. In our case, however, the bias depends on the participant, suggesting that some participants interpret the stimulus consistently in a different manner than other participants.

Explanations for the different directions of bias in the independent object motion experiments may help to interpret the pattern of results in the two groups of participants. The simple prediction of the vector sum of the self-motion and the inverse of the translation of crowd that produced a bias against the direction of crowd motion and that was followed by the majority of participants (group A, Figure 7a) is expected from an overall pooling of flow vectors when the self-motion consists of only a pure translation (Layton & Fajen, 2016b; Li, Ni, Lappe, Niehorster, & Sun, 2018; Warren & Saunders, 1995). In contrast, a bias in direction of independent motion can occur if the optic flow analysis considers a full three-dimensional motion, consisting of translational and rotational components (Li, Ni, Lappe, Niehorster, & Sun, 2018; Royden, 2002). Such a full three-dimensional model can explain biases in both directions depending on the geometry of the stimulus and the likelihood that motion vectors of the independent object can be attributed to a rotational component of self-motion (Li, Ni, Lappe, Niehorster, & Sun, 2018). Therefore, it seems to be possible that the three participants of group B (Figure 7b) attribute the translational motion of the crowd to a rotational component of their self-motion and show a bias in the direction of crowd motion while the participants of group A (Figure 7a) do not, and instead attribute the crowd translation to their self-motion and hence sum the two translational components. By keeping these two groups separate for further analysis, we will see whether individual biases in interpreting the stimuli predict similar idiosyncrasies in the only-articulation condition.

#### Biases produced by articulation of biological motion

From the analysis so far, we have seen that heading estimates in the natural articulation-plus-translation condition were unbiased. The only-translation condition produced large biases, although not consistent between participants. We have also seen that the combination of the reported headings in the only-translation and the only-articulation condition was a good predictor of the reported heading in the combined articulation-plus-translation condition. Together, this indicates that there is information in the articulation that compensates for the heading errors that result from the translation of the walkers in the crowd. Therefore, we will proceed to analyze the data of the only-articulation condition and their relationship to that of the only-translation condition.

**Figure 8.**
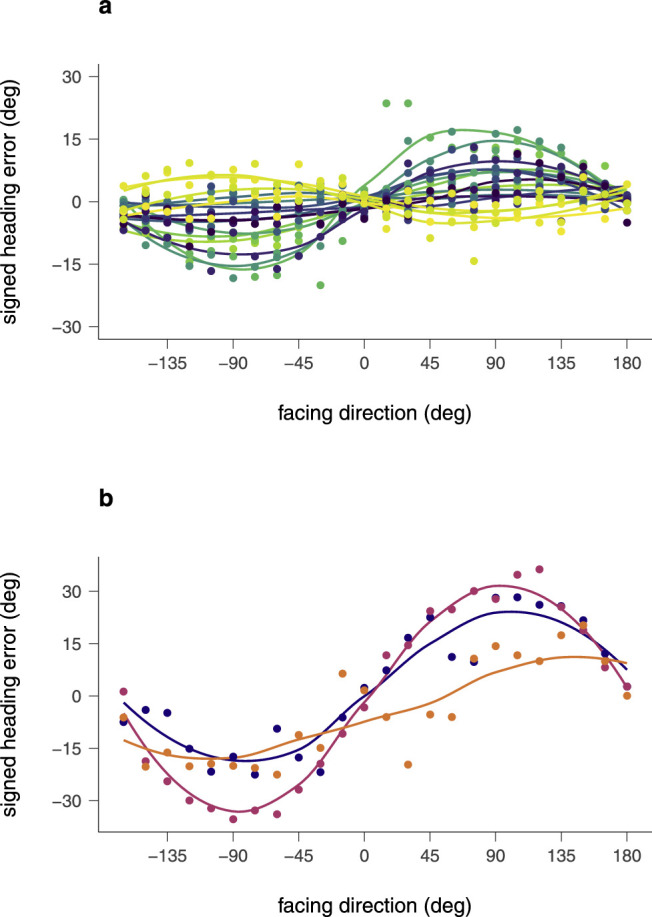
Signed heading errors of individual participants from the two groups of Figure 7 in the only-articulation condition. (a) Those individuals who showed a bias against the facing direction in the only-translation condition (group A). (b) Those individuals who showed a bias in the facing direction in the only-translation condition (group B).

Analogous to the only-translation condition, we first examined the data according to biases depending on facing direction across the full set of participants. The Friedman rank-sum test confirmed that the heading error depended on the facing direction, *A* = 7.98, *p* < 0.001; χ^2^(23, 20) = 132, *p* < 0.001. We then looked at the data separately for each of the two groups identified in the only-translation condition. We plotted data of the signed heading error of group A in the only-articulation condition in Figure 8a and the signed heading error of group B in Figure 8b.

Figure 8b shows that the three participants from group B exhibit a strong bias that is leftward for facings to the left and rightward for facings to the right. The data in Figure 8a also show strong biases in individual participants, but with different and idiosyncratic directions. For both groups, we found a significant relationship between the size of the heading error and the facing direction: for group A, *A* = 2.03, *p* < 0.001; χ^2^(23, 17) = 132, *p* < 0.001; for group B, *A* = 0.86, *p* = 0.026; χ^2^(23, 3) = 60.87, *p* < 0.001.

The finding of strong biases in the only-articulation condition is remarkable since the walkers in this condition remain fixed in place in the world, and the ambulation of their limbs, for example, the swings of the two arms, is more or less balanced between opposite directions. From a point of view of pure optical flow analysis, these ambulations should produce noise, but not in any systematic direction. Thus, pure optic flow analysis would predict a bias-free, although noisy heading estimate. Because the data instead show strong biases, we must conclude that some aspect of the articulation influences and biases heading estimation from optic flow.

Next, we analyzed the relationship between the heading estimates in the only-articulation and the only-translation condition. Within these groups and at the individual level, we tested whether facing had the same or opposite effect on bias in the two conditions.

**Figure 9.**
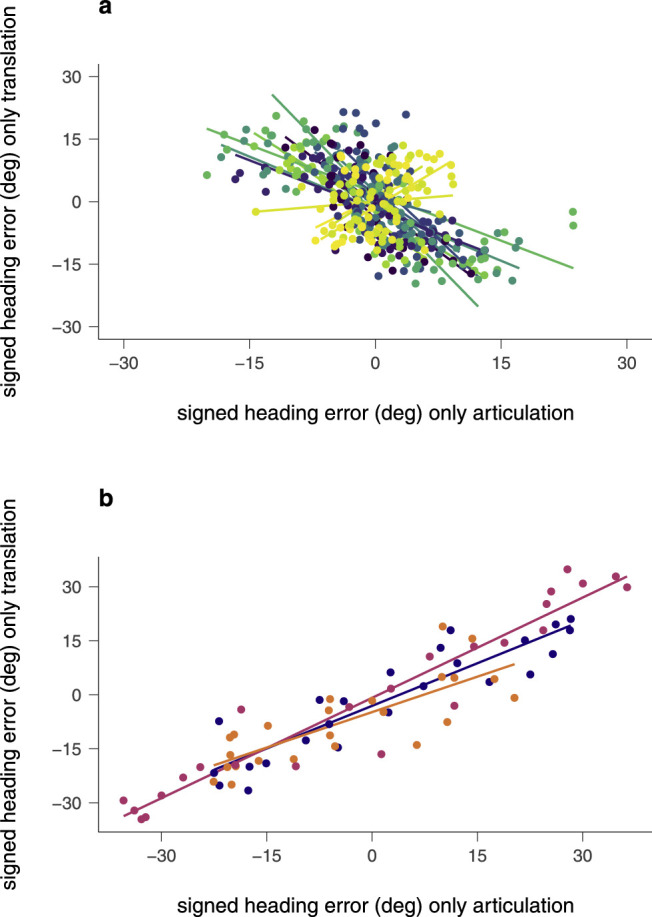
Relation of signed heading errors between the only-translation and only-articulation conditions in individual data. Each color represents an individual participant. (a) Scatterplot of individual data from group A (*n* = 17) with corresponding linear regressions. Thirteen of 17 participants displayed a negative correlation (*blue/green colors*) and four participants displayed a weak to moderate positive correlation (*yellow colors*). (b) Scatterplot of individual data from group B (*n* = three) with corresponding linear regressions. All three participants had a strong positive correlation of heading error between conditions.

Figure 9 shows linear regressions between the signed heading error in the only-articulation and the only-translation condition for each participant of the two groups. Overall, participants of group A were significantly more likely to show a bias in the opposite direction between the only-articulation and the only-translation condition, *r*_τ_ = –041, *p* < 0.001 (Figure 9a). For most subjects in this group, the negative correlation in response behavior between the two conditions also applied at the individual level. *r*_τ_ = –0.14, *p* = 0.363 to *r*_τ_ = –0.75, *p* < 0.001. Four subjects of this group, however, displayed a weakly to moderately positive correlation, *r*_τ_ = 0.14, *p* = 0.363 to *r*_τ_ = 0.44, *p* = 0.002. A positive correlation was also found for the three participants of group B (Figure 9b), both on the group level, *r*_τ_ = .76, *p* < 0.001, and on the individual level, ranging between *r*_τ_ = 0.60 and *r*_τ_ = 0.88, *p* < 0.001. We, thus, conclude that the facing dependent bias in the only-articulation condition correlates strongly with the facing dependent bias in the only-translation condition, but that the direction of the correlation differs between participants in an idiosyncratic manner.

Taken together, the results of the only-articulation condition show two remarkable findings. First, articulation in place produces a strong bias in heading perception, although the world is predominantly static, because the walkers do not translate in the world. Second, this bias can be as strong as the bias produced by walker translation, but the two conditions show positive or negative correlations in different subjects. Therefore, we must ask what aspect of biological motion produces the bias of heading perception in the only-articulation condition and how it is related to the bias in the only-translation condition.

## Experiment 2

The pattern of articulation during walking contains cues about the speed of a walker (Giese & Lappe, 2002; Masselink & Lappe, 2015; Thurman & Lu, 2016). Giese and Lappe (2002), for instance, showed that typical motion speed is encoded with its characteristic spatiotemporal structure. This means that the way someone moves on a treadmill (running vs. walking, for example) indicates the speed even when there is no physical translation. Thurman and Lu (2016) confirmed that humans use articulation as a speed cue to discriminate human actions. Further investigations by Masselink and Lappe (2015) provided evidence about how articulation combined with translation and facing contributes to accurate perception of biological form. In their experiment, participants evaluated the articulation direction (leftward vs. rightward and forward vs. backward) without considering translation. Their results showed that articulation discrimination was best when translation speed and articulation matched. Further, inconsistent translational speed impaired performance. From this finding, Masselink and Lappe (2015) concluded that translation drives the perception of articulation in the translational direction.

Biological motion can provide a reference frame onto which other position or motion perception tasks are judged (Tadin, Lappin, Blake, & Grossman, 2002). Fujimoto and colleagues showed that articulation in place produces illusory background motion opposite to the facing direction of the walker (Fujimoto, 2003; Fujimoto & Sato, 2006; Fujimoto & Yagi, 2008). It, thus, seems possible that aspects of the gait cycle of the point-light walkers imply motion of the reference frame for optic flow analysis, and that this biases heading estimates. This result predicts that the bias should not occur if the stimuli do not depict biological motion. Experiment 2 tests whether this is the case by adding a condition of nonbiological stimuli that provide similar motion signals but do not produce a percept of biological motion. In the nonbiological stimuli, the human body structure was disrupted by randomly displacing the points while the motion trajectories are kept the same (spatial scrambling; Cutting, 1981). In other respects, Experiment 2 was identical to Experiment 1. However, the facing directions were restricted to –90° and +90°, the two directions that produce the strongest bias, and 0° and 180°, two directions that produce no bias.

If the effect of articulation we observed in Experiment 1 is specific to biological motion we expect (a) that the heading error at ±90° in the natural articulation-plus-translation condition is smaller in the walker condition than in the nonbiological condition, and (b) that the bias at ±90° in the only-articulation condition occurs only with the walkers but not with the nonbiological stimuli. Moreover, we expect that heading errors in the static and only-translation conditions do not differ between the stimulus types.

### Methods

#### Sample

Fourteen participants from the University of Münster took part in Experiment 2. The data of one participant could not be used owing to technical issues during data collection. Another participant was excluded because of existing visual impairments, which he disclosed only after the experiment. Thus, the final sample consisted of 12 participants (four males, eight females). Participant age ranged from 19 to 32 years (*M* = 24.17, *SD* = 4.02). Conditions of participation remained identical to the previous experiment.

#### Setup

The experimental setup was identical to the first experiment other than that a newer version of MATLAB (version R2019b, The MathWorks) with the Psychophysics Toolbox V3 (Kleiner, Brainard, & Pelli, 2007) and the OpenGL libraries (version 2.1) add-on was used. Experiments were run on an Apple MacBook Pro (equipped with an Intel UHD Graphics 630 1536 MB).

#### Scene

Experimental details followed Experiment 1 regarding stimulus conditions and presentation, general procedure, and experimental task. Walkers and nonbiological stimuli were tested in separate experimental blocks. Within each block, stimulus combinations were randomized for each participant. The order of blocks was counterbalanced. Different from Experiment 1, the walkers appeared with only four facing directions (to the left, –90°; to the right, +90°; straight ahead toward the observer, 0°; and straight away from the observer, 180°). For the nonbiological stimuli, the starting locations for each point of the point-light walkers were assigned randomly within the area normally covered by the walker.

#### Conditions

We designed the conditions identically to Experiment 1 and measured perceived heading as a function of facing direction of the walkers in four conditions. All facing directions were combined with all four conditions, resulting in a total of 16 combinations. One block comprised all stimulus combinations five times, resulting in 80 trials. For each stimulus type, we measured two experimental blocks.

### Data analysis

For the data analysis, we concentrated on the heading error at ±90° as our previous study results suggest that there is no significant heading error at facings of 0° and 180°. Because we were interested in whether articulation and translation produce a bias in or against facing direction, and since the biases at +90° and –90° should be opposite and symmetric, we combined heading errors at +90° and –90° to compute the error in facing direction as our dependent variable. The error in facing direction is calculated from the signed heading error at –90° minus the signed heading error at 90°, or error at –90° + (–1) × error at +90°.

### Results and discussion

One participant showed an error of more than 3 standard deviations from the mean and was considered an outlier. This participant was removed from further analysis resulting in a sample size of 11. In the static condition, mean errors in facing direction were small, walker, *M* = 0.30, *SD* = 6.31; nonbiological, *M* = 0.44, *SD* = 5.72, and not different between stimulus types, *t*(109) = 0.18, *p* = 0.859, as expected.

Also as expected, the only-translation condition produced a strong bias for both stimulus types, walker, *M* = 17.40, *SD* = 13.79; nonbiological, *M* = 21.02, *SD* = 11.81, with errors being larger than in the static condition in each case, walker, *t*(109) = –11.67, *p* < 0.001; nonbiological, *t*(109) = –16.01, *p* < 0.001. Within the only-translation condition, the bias for the walkers did not differ from that of the nonbiological stimulus, *t*(109) = 2.29, *p* = 0.072.

**Figure 10.**
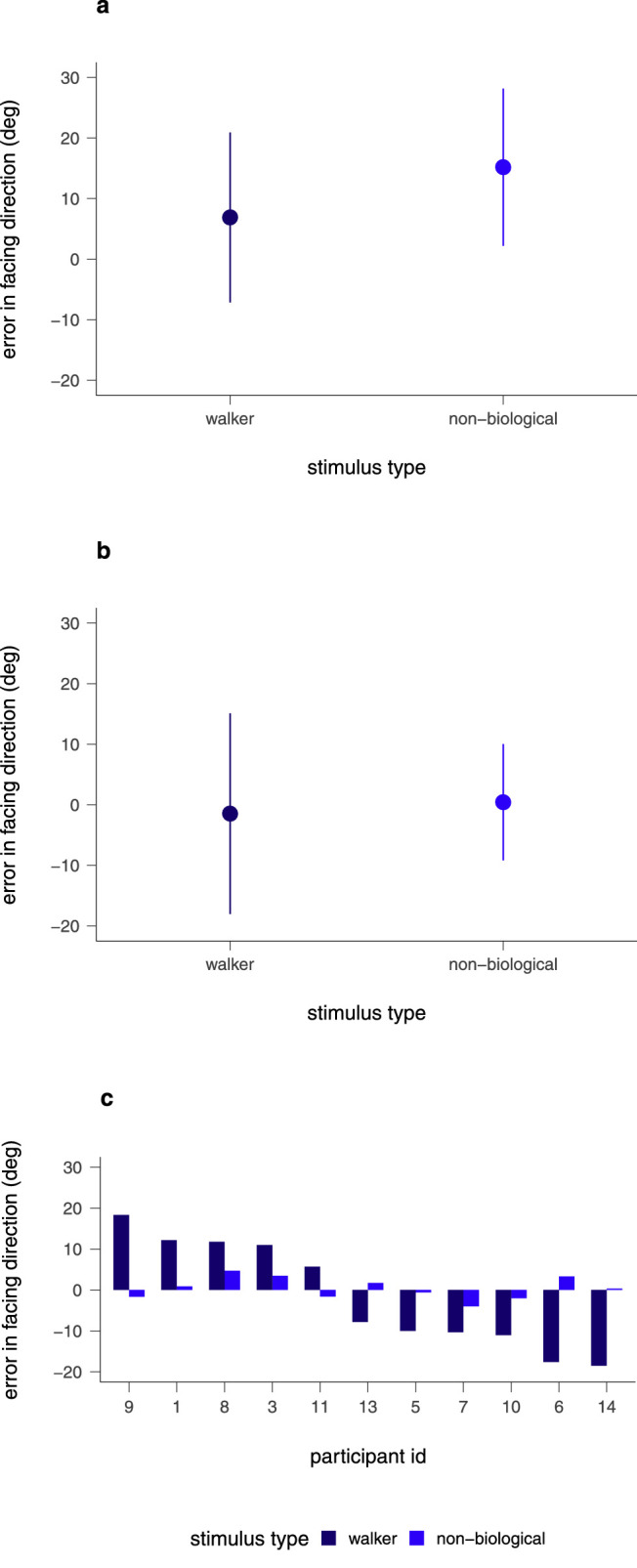
Results of Experiment 2. (a, b) Mean errors in facing direction for the normal and nonbiological walker types in the natural articulation-plus-translation condition (a) and the only-articulation condition (b). Error bars give the standard deviations of the mean. (c) Errors in facing direction in the only-articulation condition from all individual participants.

The mean errors in facing direction for the natural translation-plus-articulation condition are shown in Figure 10a. The error for the nonbiological stimulus, *M* = 15.18, *SD* = 12.99, was larger than the error for the walker, *M* = 6.89, *SD* = 14.04) (*t*(109) = 4.74, *p* < 0.001. Hence, removing biological motion removed the benefit of articulation in this condition.

In the only-articulation condition, mean errors in facing direction were small and did not differ between stimulus types, *t*(109) = 1.04, *p* = 0.299 (Figure 10b). However, the variance of the errors was much higher for the walkers than for the nonbiological stimuli, *F*(109, 109) = 2.98, *p* < 0.001. This finding suggests that individual participants might have produced large biases for the walker stimulus but, as in Experiment 1, the biases might be in idiosyncratic directions and cancel out in the average. Indeed, Figure 10c shows that this is the case by plotting the error in facing direction of each of the eleven participants individually. Biases were indeed large, up to 20°, but one-half of the participants showed biases in the facing direction, whereas the other participants showed biases against the facing direction. In contrast, errors were consistently small for the nonbiological stimuli.

The results of Experiment 2 confirm that biological motion influences heading estimation. First, the error in facing direction in the natural articulation-plus-translation condition is smaller for the walker than for the nonbiological stimulus. Second, an idiosyncratic bias in the articulation condition appears only for the walker but not for the nonbiological stimulus. Third, heading errors in the static and only-translation conditions do not differ between stimulus types.

## General discussion

We investigated errors of heading perception from optic flow when a moving observer encountered a group of walkers that faced and walked collectively in a single direction. No other environmental features were visible, and the visual motion of the walkers provided the only available information. Thus, each of the points in the scene combined the motion of the optic flow produced by observer motion with the biological motion (i.e., the translation and the articulation) of the walker. Hence, finding the proper direction of heading required to discount or remove the biological motion component and estimate heading from the optic flow component alone.

We found that observers were quite capable of doing this task with median unsigned errors not much higher than in a control condition in which the entire group stood still, and optic flow was produced only by the observer motion. The overall good performance is in line with previous research on heading through a crowd of walkers that walked in random directions (Riddell & Lappe, 2018).

Because the walkers in our experiment all faced in the same direction and, thus, had a collective direction of walking, we were also able to investigate any systematic influence of biological motion on the signed error of heading estimation, that is, the heading bias. We explored facing directions along a full circle, encompassing facing and walking toward the observer, away from the observer, toward the left and right, and in-between directions. We found that the median signed error was small and did not depend on the facing direction of the group, again suggesting that observers were, on average, surprisingly good at this task, considering the massive violation of a central assumption of optic flow analysis, the rigidity of the environment.

Specifically, median heading errors were small both when the group approached the observer (facings around 0°) and when the group receded (facings around 180°), that is, when the observer followed the group, despite the speed of the optic flow being vastly different in these two cases. In fact, in the latter case, there was much smaller relative motion between the crowd and the observer since both moved in the same direction.

In previous work, Riddell and Lappe (2017) showed that heading perception for observer movement toward a single walker was strongly biased by the walker's facing and walking direction. Because this bias was not seen in the present study, some aspects of the group motion must have helped to derive the proper heading. Perhaps, as Riddell and Lappe (2018) speculated, a single walker does not contain sufficient information to stabilize heading perception, but a group of walkers does.

The walkers in the group translated through the world and moved their limbs in the typical articulation pattern of walking. Because these two aspects of biological motion are coupled in natural locomotion (Masselink & Lappe, 2015), the articulation pattern might be helpful to estimate the translation and discount or remove the translational aspect from the combined motion pattern. To investigate this, we also studied heading perception in conditions that presented only-translation, that is, without articulation, and only-articulation, that is, with walkers walking in place, as if on a treadmill. An inferential analysis showed that the errors in the natural condition that combined articulation and translation were well-predicted by the errors in the separate only-translation and only-articulation conditions. This finding suggests that observers used articulation information from biological motion to counteract biases produced by the translation. However, these biases were not systematic but idiosyncratic.

In the only-translation condition, most observers showed a bias against the facing and walking direction of the group. Three observers, in contrast, presented an equally strong (up to 30°) bias in the opposite direction, that is, in the direction of facing and walking of the group. These biases may be related to differences in optic flow analysis because it has been shown that biases resulting from independent object motion in the optical flow occur both in the direction of and against the object motion, depending on exact parameters, for example the angle of motion of the object related to the direction of heading of the observer (Layton & Fajen, 2016b; Li, Ni, Lappe, Niehorster, & Sun, 2018; Royden & Hildreth, 1996; Warren & Saunders, 1995). The different directions of bias have been explained by the consistency of the object motion with two possible interpretations of the self-motion, one which consists of pure translational motion of the observer and one which consists of a translation in conjunction with a rotation of the eye (Li, Ni, Lappe, Niehorster, & Sun, 2018). A rightward motion of an object, for example, could be seen as an indication of either a leftward translation of the observer or a leftward rotation of the eye. Its combination with the forward motion of the observer could, thus, either produce a bias to the left (as a combination of leftward translation and forward translation) or a bias to the right, because the retinal reference frame in which the forward motion of the flow is initially encoded needs to be rotated to the right to compensate for the leftward eye rotation. In the present study, both interpretations are possible in the only-translation condition because the motion of the points is also a combination of forward motion of the observer with sideways motion of the group. In this view, one set of observers may have perceived the pure translation explanation of the stimulus, whereas a smaller set of observers might have consistently perceived the translation and rotation explanation.

Both explanations have in common, however, that heading biases are explained by the assumption that the visual system treats all image motion indiscriminately, as if resulting from self-motion in a rigid world, consistent with several previous studies (Li, Ni, Lappe, Niehorster, & Sun, 2018; Riddell & Lappe, 2017; Riddell, Li, & Lappe, 2019). In the only-translation condition, this result is to be expected because the stimulus does not contain any information about the movement of the group itself. Hence, the visual system has no way of knowing how the group moved. In the natural translation-plus-articulation condition, in contrast, articulation provides information about the movement of the group (Giese & Lappe, 2002; Masselink & Lappe, 2015; Thurman & Lu, 2016). The finding in Experiment 1 that heading perception is largely bias free in this condition showed that the visual system used the information in the articulation pattern. Moreover, Experiment 2 showed that the use of the articulation pattern is specific to biological motion perception since the benefit of articulation is not seen in the nonbiological stimuli.

The influence of articulation on heading estimates is most directly seen in the only-articulation condition. Because the walkers remained in place in this condition, the observed bias must result directly from the articulation. Like in the only-translation condition, the bias was idiosyncratic, with some participants showing a consistent bias in, and others against, the facing direction. These biases, too, are specific to biological motion because they did not occur for the nonbiological stimuli in Experiment 2. They are not directly linked with the bias in the only-translation condition, however. For some participants, the bias is in the opposite direction from that in the only-translation condition, suggesting that the articulation can provide a compensatory effect on the bias produced by the translation. For other participants, the bias is in the same direction as in the only-translation condition. The inconsistencies in the idiosyncratic bias in these two conditions point to possible differences in the mechanisms by which translation and articulation influence heading estimation.

One clear difference between the only-translation and the only-articulation conditions is that the former includes translational motion of the group that is combined with the flow from the observer movement, whereas the latter does not. However, articulation in place, as in the only-articulation condition, is known to produce illusory background motion in the opposite direction of the walker's facing (Fujimoto, 2003; Fujimoto & Sato, 2006; Fujimoto & Yagi, 2008). This illusion implies that articulation in place may produce an apparent shift of the reference frame in which the optic flow is encoded (i.e., the retina) (Tadin, Lappin, Blake, & Grossman, 2002), thereby producing a bias either against or in the direction of the illusory motion, respectively. Alternatively, participants might infer from the articulation pattern an implied translation of the walker that did not exist in the stimulus. This finding implied that translation could also be erroneously interpreted as self-rotation. In either case, the influence of articulation on heading estimation is not a direct contribution to optic flow analysis, which takes place in retinal coordinates and is based on retinal motion, but rather an independent modification of the reference frame, which takes place in the transformation from retinal to allocentric coordinates.

Such an effect of articulation on the reference frame of heading estimation may also explain the difference between our present findings and those of Riddell and Lappe (2018). Although both studies agree that heading errors are smaller for the combination of translation and articulation than for translation alone, that is, that articulation is helpful in estimation of heading toward a crowd, the articulation benefit in the study of Riddell and Lappe (2018) was also seen for inverted and nonbiological scrambled walkers. Riddell and Lappe (2018), therefore, concluded that the benefit of articulation was not specific to biological motion. They, furthermore, provided evidence that the articulation benefit was based on brief periods in the natural (translation plus articulation) walking cycle in which some points, such as the feet when touching the ground, remained stable in the world, allowing a brief glimpse of the rigid environment. Crucially, this invariant cue to heading perception is also present when the stimuli are presented spatially scrambled, because it is contained in the trajectories of the individual hinge points, not in the overall form of the walkers.

This cue is available, also, in the stimuli of the present study and may have provided part of the benefit of the articulation. However, in addition to Riddell and Lappe (2018), the present study showed a specific benefit of articulation for the normal walkers over the nonbiological scrambled stimuli. This additional benefit may derive from the fact that the entire group in the present study always faced and moved in a single direction while in the study of Riddell and Lappe (2018), the walkers in the crowd faced and moved in individual random and overall balanced directions. In that case, it is unlikely that the group produces a coherent backscroll illusion or a coherent implied change of reference frame. Likewise, the random directions of the crowd constituents make a coherent bias in the only-translation condition improbable.

In this view, it is also possible that the influence of biological motion only occurs if the walkers are perceived as a group or a common gestalt. Group motion is a prime example of gestalt perception, known as the law of common fate, and recent research has shown that groups of coherently walking point-light walkers give rise to ensemble coding mechanisms (Sweeny, Haroz, & Whitney, 2013; Whitney & Yamanashi Leib, 2018). It would be interesting to further explore the effect of ensemble perception in self-motion toward crowds.
